# Alteration of Histone Acetylation Pattern during Long-Term Serum-Free Culture Conditions of Human Fetal Placental Mesenchymal Stem Cells

**DOI:** 10.1371/journal.pone.0117068

**Published:** 2015-02-11

**Authors:** Yongzhao Zhu, Xumei Song, Fei Han, Yukui Li, Jun Wei, Xiaoming Liu

**Affiliations:** 1 Key Laboratory of the Ministry of Education for Conservation and Utilization of Special Biological Resources in Western China, Ningxia University, Yinchuan, 750021, China; 2 Human Stem Cell Institute of General Hospital, Ningxia Medical University, Yinchuan, 750004, China; 3 School of Laboratory Medicine, Ningxia Medical University, Yinchuan, 750004, China; The University of Tennessee Health Science Center, UNITED STATES

## Abstract

Increasing evidence suggests that the mesenchymal stem cells (MSCs) derived from placenta of fetal origin (fPMSCs) are superior to MSCs of other sources for cell therapy. Since the initial number of isolated MSCs is limited, *in vitro* propagation is often required to reach sufficient numbers of cells for therapeutic applications, during which MSCs may undergo genetic and/or epigenetic alterations that subsequently increase the probability of spontaneous malignant transformation. Thus, factors that influence genomic and epigenetic stability of MSCs following long-term expansions need to be clarified before cultured MSCs are employed for clinical settings. To date, the genetic and epigenetic stability of fPMSCs after long-term *in vitro* expansion has not been fully investigated. In this report, alterations to histone acetylation and consequence on the expression pattern of fPMSCs following *in vitro* propagation under serum-free conditions were explored. The results show that fPMSCs maintain their MSC characteristics before they reached a senescent state. Furthermore, acetylation modification patterns were changed in fPMSCs along with gradually increased global histone deacetylase (HDAC) activity and expression of HDAC subtypes HDAC4, HDAC5 and HDAC6, as well as a down-regulated global histone H3/H4 acetylation during *in vitro* culturing. In line with the acetylation alterations, the expression of oncogenes Oct4, Sox2 and TERT were significantly decreased over the propagation period. Of note, the down-regulation of Oct4 was strongly associated with changes in acetylation. Intriguingly, telomere length in fPMSCs did not significantly change during the propagating process. These findings suggest that human fPMSCs may be a safe and reliable resource of MSCs and can be propagated under serum-free conditions with less risk of spontaneous malignancy, and warrants further validation in clinical settings.

## Introduction

Mesenchymal stem cells (MSCs) have been investigated extensively as one of the most promising cell types for therapeutic applications. MSCs isolated from a wide range of tissues and organs, including bone marrow, adipose tissue, umbilical cord, amniotic membrane, and placenta have been investigated in experimental and/or clinical settings [1–3]. Apart from an advantage in maintenance of stemness, MSCs derived from fetal origins (fMSCs) have recently been demonstrated to possess properties of higher capacities of proliferation, specific lineage differentiation and immunomodulation, as compared to MSCs isolated from adult tissues [4–9]. In respect to fMSCs, fetal placental mesenchymal stem cells (fPMSCs) have attracted more attention for both research and clinical applications, owing to a superior potential for immunomodulation and tissue repair while avoiding many major ethnical concerns of other sources [10,11].

Like MSCs harvested from other tissues, fPMSCs also must be expanded *in vitro* in order to reach sufficient cell numbers for pre-clinical and/or clinical applications. However, during *in vitro* propagation MSCs may acquire genetic and/or epigenetic mutations and subsequently may undergo spontaneously tumorigenic transformation [12–14]. In this regard, increasing evidence has suggested that epigenetic modifications, such as DNA methylation and histone acetylation, could occur in progeny of MSCs during an *in vitro* culturing process [10,15–17]. Over a long-term culture period human MSCs that have acquired methylation modifications in promoter regions within tumor suppressor genes, HIC1 and RassF1A, exhibited cancer stem/initiating cell like properties [18]. The notion that malignant transformation of MSCs during *in vitro* expansion remains alarming due to early studies from two other groups, they reported that culturally expanded murine MSCs showed potential for tumorigenesis including accumulation of chromosomal abnormalities, aberrant gene expression, elevation of telomerase activity, and malignant transformation [19,20]. Several lines of study have demonstrated that MSCs derived from both human and murine tissues can acquire a series of genetic and/or epigenetic alterations during a course of long-term culture, but these studies provided no evidence of MSC-transformed malignancy in immunodeficiency mouse models [21–23]. Nevertheless, these studies suggest that genetic/epigenetic alternations may impart a potential for malignant transformation, and the safety of genetic/epigenetic modifications in MSCs thus needs to be adequately investigated in multiple aspects and clarified prior to the clinical use of culturally expanded MSCs [10,15–18]. To date, there is no solid evidence on whether histone acetylation patterns contribute to spontaneous malignant transformation in cultured MSCs, although an acetylation-altered gene expression profile was observed in cultured MSCs [24]. Our group also recently demonstrated that fPMSCs acquired methylation modifications but failed to undergo malignant transformation over an *in vitro* culture process in serum-free conditions [10], but acetylation modifications remained elusive. The objective of this study is to interrogate potential changes in histone acetylated mutations in fPMSCs during prolonged *in vitro* expansion in serum-free medium by assessing changes in the capacity for proliferation, the activity of histone deacetylases (HDACs), global histone H3/H4 acetylation alterations, and the expression of oncogenes modified by histone acetylation at different passages of fPMSCs.

## Material and Methods

### Ethics statement

Human placentas were collected with a protocol approved by the Ethics Committee for the Conduct of Human Research at Ningxia Medical University (ECCHRNMU20110307MSC-1). Written consent was obtained from each individual (mother) according to the Ethics Committee for the Conduct of Human Research Protocol. All participants provided written, informed consent for the publication of data. The Ethics Committee for the Conduct of Human Research at Ningxia Medical University approved this study (ECCHRNMU2013RP0196).

### Isolation and *in vitro* expansion of fPMSCs using a serum-free medium

Five human full-term placental tissues (3 males and 2 females) were obtained from healthy mothers at the time of routine elective caesarean section in the General Hospital of Ningxia Medical University. Informed consent was obtained from each mother prior to delivery. Fetal P-MSCs (fPMSCs) were isolated and origin-verified genetically as described previously [25]. Cells were cultured in serum-free medium composed of MesenCult-XF Basal Medium plus MesenCult-XF Supplement (STEMCELL Technologies Inc., Grenoble, France) supplemented with 50 μg/mL of gentamycin (Invitrogen, Carlsbad, CA, USA). All cultures were maintained at 37°C in a humidified incubator with 5% CO_2_. The cultures were dissociated and passaged using the MesenCult-ACF Dissociation Kit (STEMCELL Technologies Inc, Grenoble, France) when cells reached about 90% confluency

### Flow cytometry

The immunological characterization of fPMSCs was analyzed by flow cytometry on a FACS Calibur flow cytometer per the manufacturer's recommendations (BD Biosciences, San Diego, CA, USA). Single-cell suspensions were obtained after the cells were detached with MesenCult-ACF Dissociation Kit. Cells were resuspended at 1 10^6^ cells per 100 μL blocking buffer containing one of the following fluorescent dye-labeled monoclonal antibodies, and incubated for 30 min at room temperature: IgG1-PE, CD34-PE, CD73-PE, IgG2a-FITC, CD14-FITC, CD45-FITC, CD90-FITC, CD105-FITC or HLA-DR-FITC (BD Pharmingen, Franklin Lakes, NJ, USA). Following extensive washing, the cells were resuspended in 500 μL of PBS and phenotyped by flow cytometry.

### Cell proliferation assay

The proliferative capacity of cells was assessed by an MTT (methyl thiazoly tetrazolium) assay. fPMSCs were seeded and cultured in 96-well plates at a density of 0.2×10^4^/well, and proliferation of cells was measured by an MTT assay on days 1, 2, 3, 4, 5, 6, 7 and 8 post-seeding. Briefly, 20 L of 5 mg/mL MTT solution was added to each well and incubated at 37°C for 4 h, after each time the medium was replaced with 150 L of DMSO and incubated for an additional 10 min. The capacity for cell proliferation was then determined on a Microplate spectrophotometer at 490 nm (Bio-Rad Laboratories Inc., Hercules, USA). For each time point the readouts of each well was collected, and the means from five wells of fPMSCs from individual donors were used for analysis. The experiment was performed in triplicate for cells from each donor.

### Senescence-associated β-galactosidase staining assay

The population of senescent cells was determined by assessing the cellular β-galactosidase activity as described elsewhere using a Senescence β-galactosidase Staining Kit per manufacturer’s instructions (Cell Signaling Technology, Danvers, MA, USA). Briefly, fPMSCs at designated passages were seeded in 6-well plates at a density of 1 10^5^ cells/well and cultured to a confluence of 80%. The cells were then fixed with 1 Fixative Solution for 15min at room temperature, washed with 1 PBS three times after the fixation, and then incubated with 1 mL of β-galactosidase Staining Solution at 37°C overnight. Representative images were acquired, and β-galactosidase positive (blue) cells were counted in 10 random fields for each well under a phase-contrast microscope (magnification at 100×). The mean of β-galactosidase positive (blue) cells for each field was calculated. The experiment was repeated three times for each group.

### RNA Isolation and quantitative reverse transcription-PCR (qRT-PCR)

Total RNA was isolated from fPMSCs with Trizol Reagent (Invitrogen, Carlsbad, CA, USA). First-strand cDNA was synthesized with the Revert Aid First Strand cDNA Synthesis Kit using 4 g of total RNA (Thermo Fisher Scientific, San Diego, CA) and stored at -20℃ till used. qRT-PCR was performed on a Bio-Rad iQ5 lightcycler using the DyNAmo Capillary SYBR Green 2-Step qRT-PCR Kit (Thermo Fisher Scientific Inc, MA, USA). The thermal cycling conditions were 95˚C for 10 min, followed by 40 cycles of 15 s at 95˚C and 1 min at 60˚C. Relative expression was calculated as previously described using real-time PCR efficiencies and the crossing point deviation of unknown sample *vs* control using a ΔΔ Ct method [26]. Primer sets used in this study are listed in Table 1.

**Table 1:** Primer sets for Real-Time qPCR.

Gene	Forword (5’-3’)	Reverse (5’-3’)
HDAC1	AGCCAAGAGAGTCAAAACAGA	GGTCCATTCAGGCCAACT
HDAC2	GCTCGATGTTGGACGTATGAGAC	ACCTCCTTCACCTTCATCCTCAG
HDAC3	GGTGGTGGTGGTTATACTGTC	TGGATGAAGTGTGAAGTCTGG
HDAC4	ACACTCGGCTCTTCTCCAC	TGCTCGCTTGCTGACTCC
HDAC5	TCCTCCTTCCTCTTGGTCTC	CTGGTTCTGTTACTCCTCCTG
HDAC6	CAACTGAGACCGTGGAGAG	CCTGTGCGAGACTGTAGC
HDAC7	TGAAGAATGGCTTTGCTGTG	CACTGGGGTCCTGGTAGAAA
HDAC8	GATGGGGAGCGGTGATAGTGTC	TTCCGCAGCCACCTTCCAG
HDAC9	GTTCCCTTACATCCTCAGTCTC	TGGTATTGCTTCTGCTTCTCC
HDAC10	GGTATAGCAGCCACTCCAG	GTTCAGCCACAGACTCCTC
HDAC11	CGGACCCAGACAGGAGGAAC	GCTTCTGTGCCTATGCGGAC

### Histone deacetylase (HDAC) activity assay

The total HDAC activity of fPMSCs derived from three donors was determined using EpiQuik HDAC Activity/Inhibition Assay Kits (Epigentek, Brooklyn, NY, USA) according to the manufacturer’s protocol. Briefly, whole nuclear protein was extracted using an EpiSeeker Nuclear Extraction Kit (Abcam plc, Cambridge, UK). The nuclear extraction (NE) was then incubated with HDAC specific substrate for 1 h at 37℃. The capture antibody was added to the above reaction mixture and incubated for 1 additional hour, followed by the addition of a detection antibody and incubated for 30 min at room temperature. Total HDAC activity (OD/h/mL) was determined by assessing the absorbance at 450 nm and calculated using the following formula: [OD (control—blank)—OD (sample—blank)]/reaction time (hour) × dilution of sample. This assay determined total HDAC activity and was not gene-specific or specific for any particular HDAC.

### Global histone H3/H4 acetylation assay

Histone proteins from fPMSCs derived from three individuals at P3 and P8 were extracted using the EpiSeeker Histone Extraction Kit (Abcam). Protein concentration was determined with a bicinchoninic acid (BCA) assay (BOSTER, Wuhan, China). The acetylation level of global histone H3 and H4 was quantified using the EpiQuik total histone H3/H4 acetylation assay kit (Epigentek) according to the manufacturer’s protocol. Briefly, acetylated histones were detected with a colorimetric conversion assay and the OD at 450 nm was obtained. The percentage of histone H3/H4 acetylation in total histone proteins was then calculated using the following formula: OD (sample—blank) / OD (untreated control—blank) × 100%.

### Chromatin immunoprecipitation (ChIP) assay

Chromatin immunoprecipitation (ChIP) assay was performed using a ChIP Assay Kit (Beyotime Institute of Biotechnology, Haimen, China) per manufacturer’s protocol. Briefly, in order to establish a cross-link between targeted proteins and relevant genomic DNA regions, cells were treated with 1.0% formaldehyde in PBS for 10 min at 37°C, followed by the addition of 125 mM glycine to quench unreacted formaldehyde for additional 5 min at room temperature. Then the cells were pelleted and resuspended in lysis buffer. Samples were then subjected to sonication to shear cross-linked DNA to 200–1000 base pairs in length. Sheared DNA samples were incubated with an Anti-acetyl-lysine antibody (Millipore, Danvers, MA, USA) overnight at 4°C. The reaction mixture was then incubated with Protein A+G Agarose/Salmon Sperm DNA for 1 h at 4°C for immunoprecipitation. Following extensive washing, the cross-linked Protein/DNA complexes were eluted from the beads and cross-linking was reversed to free DNA from proteins. The DNA was then purified using the Wizard SV Gel and PCR Clean-up system (Promega Corp., Madison, WI, USA) prior to PCR amplification of several genes of interest. The primer sets for PCR were as follows [24]: Oct4, forward: 5’-CTTCCACAGACACCATTGCC-3’, reverse: 5’-AGTCCCACCCACTAGCCTTG-3’; Sox2, forward: 5’-AGTTGGACAGGGAGATGGC-3’, reverse: 5’-AACCTTCCTTGCTTCCACG-3’; TERT, forward: 5’-GGCTCCCAGTGGATTCGC-3’, reverse: 5’-GGAGGCGGAGCTGGAAGG-3’. The acquired data were further analyzed using the Percent Input Method per manufacturer’s instruction (Invitrogen).

### Telomere length assessment

Genomic DNA was extracted from fPMSCs using a QIAamp DNA Mini Kit (Qiagen, Valencia, CA, USA) and stored at -20℃ untill used. Relative length of telomeres was determined using a monochrome multiplex quantitative PCR assay as described by Cawthon with minor modifications [27]. In brief, telomere primers for the telomeres of telg and telc, endogenous single copy gene control albumin of albu and albd were first synthesized. The primer sequences were as follows: Telg (5’-ACACTAAGGTTTGGGTTTGGGTTTGGGTTTGGGTTAGTGT-3’), telc (5’-TGTTAGGTATCCCTATCCCTATCCCTATCCCTATCCCTAACA-3’), albu (5’-CGGCGGCGGGCGGCGCGGGCTGGGCGGaaatgctgcacagaatccttg-3’) and albd (5’-GCCCGGCCCGCCGCGCCCGTCCCGCCGgaaaagcatggtcgcctgtt-3’). PCR reactions for generating a standard curve was included for each experiment using a 96-well PCR reaction plate. Standard curves for both telomere and albumin were generated using a dilution series of reference DNA with a 81-fold range (the amount of DNA in five dilutions was 60 ng, 30 ng, 10 ng, 6.7 ng and 0.74 ng). PCR was performed on a Bio-Rad iQ5 lightcycler using DyNAmo Capillary SYBR Green 2-Step qRT-PCR Kit (Fisher Scientific Inc, MA, USA). The thermal cycling profile was: 1 cycle of 15 min at 95°C; 2 cycles of 15 s at 94°C, 15 s at 49°C; 32 cycles of 15 s at 94°C, 10 s at 62°C, 15 s at 74°C with signal acquisition, 10 s at 84°C, and 15 s at 88°C with signal acquisition. A readout at 74°C gave the Ct value of telomeres, and the Ct values at 88°C were for the single copy gene (scg albumin). The relative amounts of telomere and albumin could be quantified with their respective standard curves. The average ratio of T (telomere) to S (single copy gene of albumin) is expected to be proportional to the average telomere length of cells. All experimental DNA samples were assayed in triplicate.

### Statistical analysis

All statistical analyses were performed using SPSS statistical analysis software (SPSS Inc., Chicago, USA). Results were expressed as Mean ± SD for illustration. Comparisons of mean values among the passages were analyzed by using a Tukey multiple-comparison test [10,17]. Wherever a *P*<0.05 was considered a significant difference.

## Results

### Characteristics of fPMSCs during *in vitro* expansion in serum-free medium

**Figure 1:**
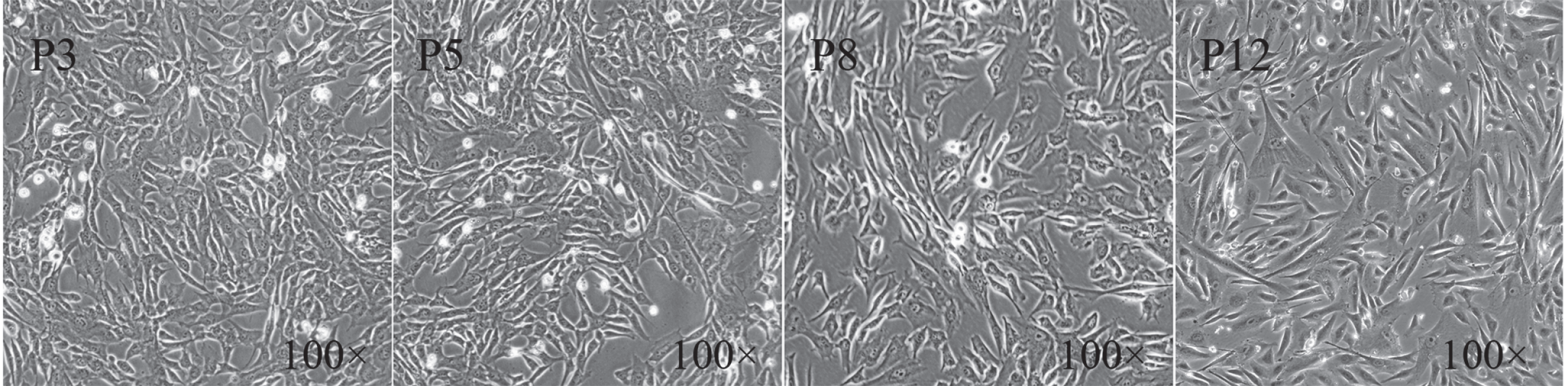
The morphology of fPMSCs in long-term cultures in serum-free medium. fPMSCs cultured in serum-free medium at passage 3 (P3), P5, P8 and P12 from five different donors were examined, fPMSCs displayed a spindle-like morphology. After P8 a population of cells with abnormally large morphologies increased with passage number.

**Figure 2:**
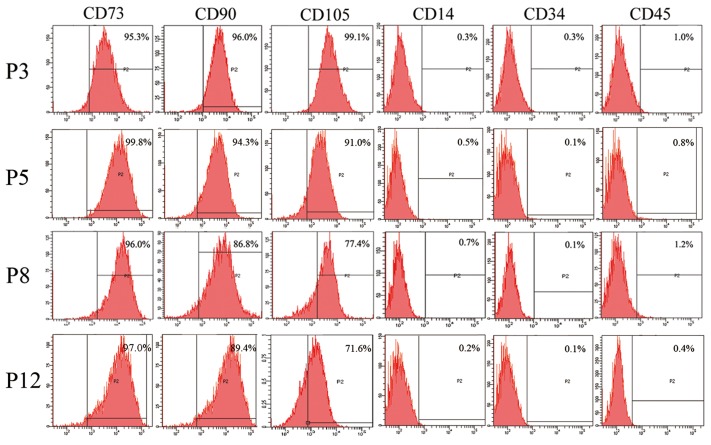
Immunophenotype of cultured fPMSCs analyzed by flow cytometry. Typical surface markers for MSCs were analyzed at P3, P5, P8 and P12 of cultured fPMSCs. All cells from the indicated passages expressed typical MSC phenotypes by flow cytometric analysis, but the number of CD105-positive cells decreased with passage number.

The characteristics of MSCs have been extensively investigated by defining their typical morphology, surface markers and differentiation potential. In our study, there was no significant difference in morphology and phenotype of fPMSCs cultured in serum-free medium during the first 8 passages. fPMSCs from all tested donors shared a similar spindle-like shape, and entered a slow-growth state with strikingly increased cellular volume at P8 onwards (P10 and P12) (Fig. 1). All tested fPMSCs expressed typical MSCs surface markers CD73, CD90 and CD105 but lacked the expression of hematopoietic cell markers CD14, CD45 and CD34 (Fig. 2). Interestingly, as seen in our previous study [10], the frequency of CD105-positive cells declined gradually during cultural expansion until P13 at which point we concluded experimentation. These results indicate that fPMSC characteristics are sustained in this type of serum-free medium during a process of propagation but we did not collect passages past P8.

### Growth kinetics of fPMSCs during expansion *in vitro*

**Figure 3:**
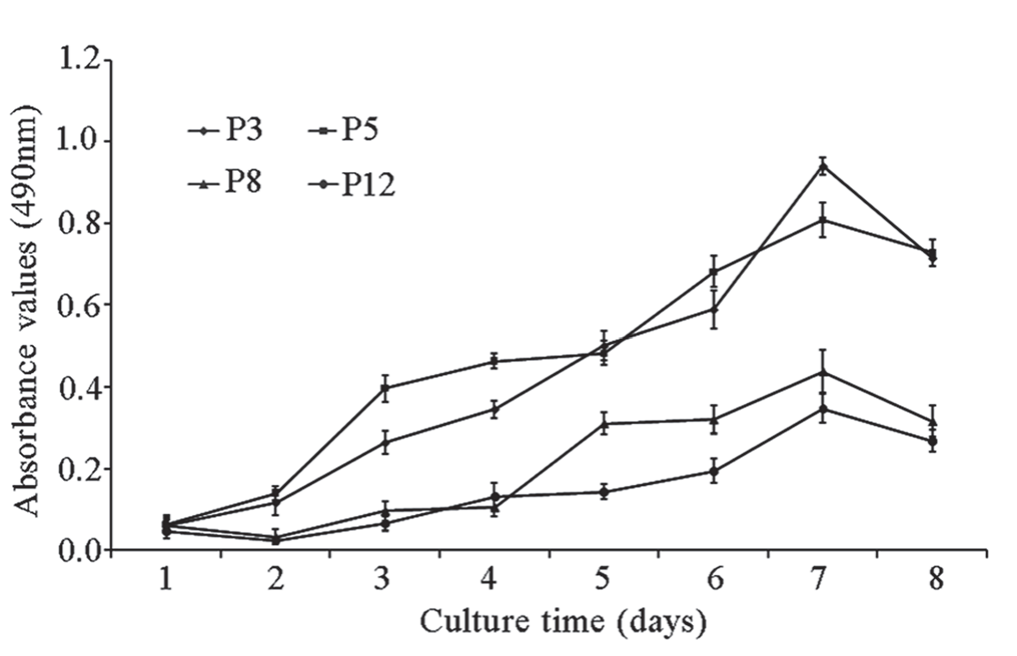
Cell proliferation analyzed by an MTT assay. Proliferative potential of fPMSCs from three donors cultured at P3, P5, P8 and P12 were seeded in culture plates in serum-free medium and assessed with an MTT assay. The proliferation rate notably decreased from P8 onward compared to earlier passages. Mean proliferative capacities for each passage number was analyzed by using a Tukey multiple-comparison test (*P*<0.05).

**Figure 4:**
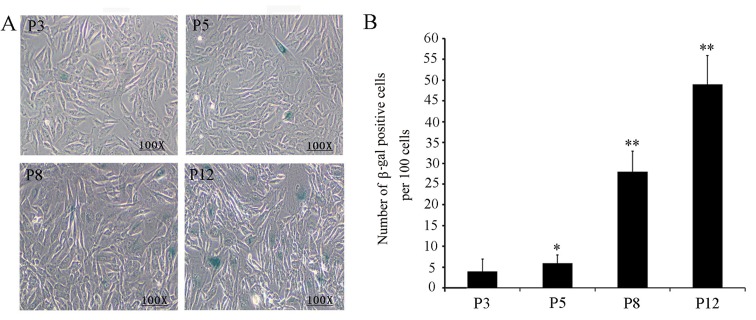
Senescent cells detected by β-galactosidase staining assay. fPMSCs derived from different donors were cultured in serum-free medium to the indicated passage and stained for β-galactosidase activity to appraise the level of senescence. β-galactosidase positive cells represent senescent cells. Compared to fPMSCs at P3, more β-galactosidase staining (blue) was observed in P8 cells and continues to increase by P12. A. Representative images of β-galactosidase staining for cells at indicated passages (magnification at 100). B. A quantitative analysis of β-galactosidase positive cells for fPMSCs at P3, P5, P8 and P12. Compared to P3 fPMSCs, **: *P*<0.01 (N = 3, student *t-test*).

We then measured the proliferative capacity of fPMSCs at different passages up to P12 using an MTT assay. In comparison with cells at P3 and P5, the proliferative capacity of fPMSCs at P8 and P12 had significantly declined (*P* <0.05). Intriguingly, a dramatic decrease in cell proliferation initiated at P8 in all tested donors (Fig. 3). Of note, fPMSCs started to enter a senescent phase at P8, this finding was supported with more abundant β-galactosidase-positive, senescent cells in later passages of cultures (P8, P10 and P12) relative to cells in an earlier passage (P3 and P5) (Fig. 4).

### Enhanced global HDAC activity of fPMSCs during a long-term *in vitro* culture

**Figure 5:**
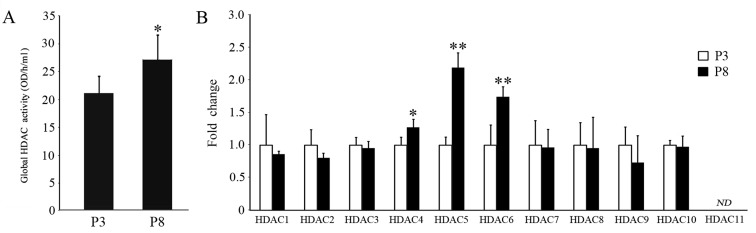
Enzymatic activity and expression of HDACs in fPMSCs. A. Global HDAC activity of fPMSCs was assessed using EpiQuik HDAC Activity/Inhibition Assay Kits. In contrast to P3 cells, the enzymatic activity of HDACs in P8 fPMSCs was dramatically decreased (P<0.05). B. The expression level of canonical HDAC family members was measured by RT-qPCR. Relative expression of HDAC4, HDAC5 and HDAC6 was significantly up-regulated in P8 fPMSCs compared to P3 cells (P<0.05). Data represent triplicate experiments with three individual donors. Relative expression was calculated using the ΔΔ Ct method against an internal GAPDH control. Fold change in gene expression in P8 cells was normalized to donor-matched cells at P3. Comparisons of mean values between P3 and P8 cells were analyzed by using a student *t-test*. Compared with P3 cells, **: P<0.01, *: P<0.05, N = 9 (triplicated experiments from three donors).

To investigate whether fPMSCs acquired acetylation modifications during long-term *in vitro* cultures under serum-free conditions, the global activity and gene expression of HDAC from fPMSCs at P3 and P8 were determined by an enzymatic assay and a qRT-PCR assay. Results from the enzymatic assay showed that global HDAC activity of fPMSCs at P8 was significantly higher compared to cells at P3 (Fig. 5A). Surprisingly, results from a qRT-PCR assay indicated that the expression of the majority of HDAC family members was not significantly altered during the expansion process, with the exception of HDAC4, HDAC5 and HDAC6, which were expressed in greater abundance in P8 fPMSCs relative to P3 cells (Fig. 5B). Interestingly, the expression of HDAC2 and HDAC9 did not change during long-term culture (Fig. 5B). These data suggest that the HDAC members, HDAC4, HDAC5 and HDAC6 may be the main contributors for enhancing global HDAC activity and may play a key role in the epigenetic regulation of biological functions in fPMSCs during cultural propagation. These results also imply that the mechanism for down-regulating the expression of certain genes in fPMSCs during *in vitro* expansion in serum-free medium might be through a epigenetic acetylation of relevant histones.

### Down-regulation of global histone H3/H4 acetylation during *in vitro* expansion of fPMSCs

**Figure 6:**
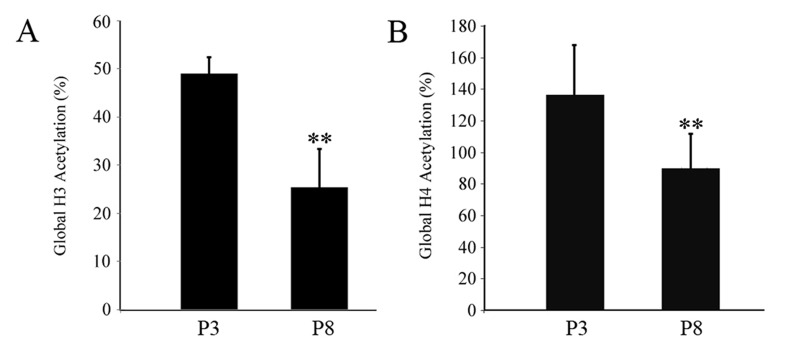
Global acetylation status of histones H3 and H4 in fPMSCs. Total histone proteins from fPMSCs at P3 and P8 were extracted, and the global acetylation levels of histones H3 and H4 were identified using an EpiQuik total histone H3/H4 acetylation assay kit. Global acetylation of both histone H3 (A) and histone H4 (B) was significantly reduced in P8 fPMSCs compared to P3 cells. Compared with P3 cells, **: P<0.01, n = 3.

Given that extended culturing in serum-free medium led to an enhanced global HDAC enzymatic activity and the increased expression of several HDAC members in fPMSCs, we interrogated the functional impact of these changes by evaluating global histone H3/H4 acetylation in both P3 and P8 fPMSCs. Consistent with global HDAC enzymatic activity, acetylation levels of both histone H3 and histone H4 were significantly reduced at P8 fPMSCs compared to P3 cells (Fig. 6A and 6B). This result implies that non-specific HADC activity in late-passage fPMSCs could be attributed to acetylation modifications.

### Genes regulated by histone modifications in cultured fPMSCs

**Figure 7:**
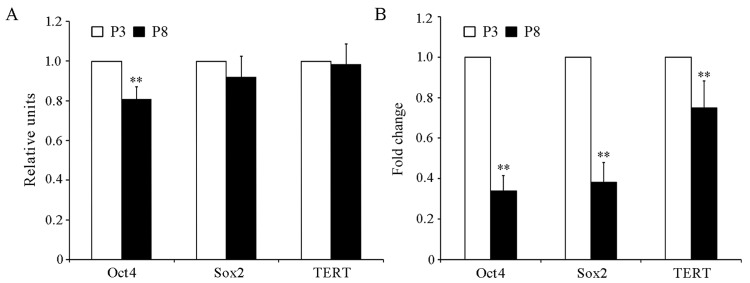
Oncogene expression and histone acetylation patterns in fPMSCs after long-term culturing. Oct4, Sox2, and TERT transcript level was determined by RT-qPCR, and the status of histone acetylation in each gene’s promoter region was detected by ChIP. A. Repressed expression of Oct4, but not Sox2 or TERT can be attributed to modulations in promoter histone acetylation. B. Oct4, Sox2, and TERT mRNA in P8 fPMSCs was significantly down-regulated, in comparison with P3 cells. Compared with P3 cells, *: P<0.05, **: P<0.01, N = 9.

We next sought to investigate whether alterations to the histone acetylation pattern were relevant to driving oncogenic potential of MSCs. We interrogated histone acetylation status at the promoters of octamer-binding transcription factor 4 (Oct4), SRY (sex determining region Y)-box 2 (Sox2) and telomerase reverse transcriptase (TERT) by ChIP assay and qRT-PCR. ChIP revealed that histone acetylation in the Oct4 promoter was significantly reduced in P8 fPMSCs relative to P3 cells (*P* < 0.05), but there was no significant difference in histone acetylation in the Sox2 or TERT gene promoters (Fig. 7A). Concurrent with the ChIP assay results, Oct4 expression was also significantly reduced in later passage fPMSCs by qRT-PCR (Fig. 7B). Unexpectedly, both Sox2 and TERT transcript abundance was reduced in P8 cells relative to P3 cells, implying that molecular mechanisms other than histone modifications might be involved in regulating Sox2 and TERT mRNA. Given that Oct4, Sox2, and TERT are fundamental regulators of self-renewal and differentiation, and only limited histone acetylation modifications of above oncogenes in the fPMSCs propagated in serum free medium may imply that they are less potential to develop spontaneous tumors.

### Reduced telomere length in late-passage fPMSCs

**Figure 8:**
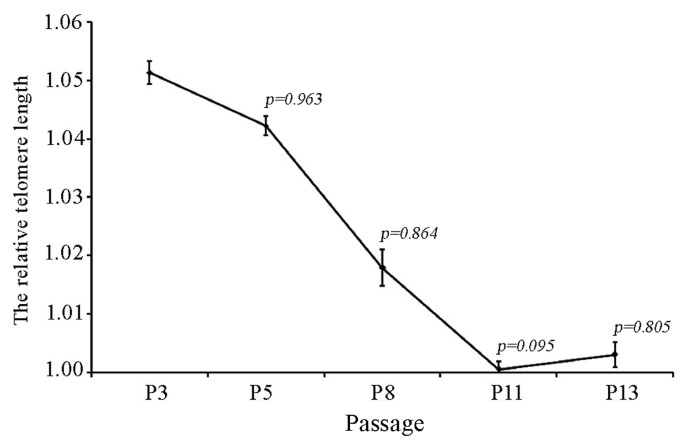
Analysis of telomere length in cultured fPMSCs. Relative telomere lengths of fPMSCs at P3, P5, P8, P11 and P13 were assessed by a qPCR assay based on Cawthon’s method. Despite a the observation that telomere length gradually shortened over time, relative telomere length did not significantly change over the course of *in vitro* expansion. P-values were obtained by comparing cells at the indicated passage number with P3 cells using a student *t-test*.

The effects of *in vitro* expansion on telomere length in cultured fPMSCs was evaluated by monochrome multiplex quantitative PCR [27]. As expected, mean telomere length in fPMSCs at later passages (P8, P11 and P13) was marginally reduced relative to cells harvested at an earlier passages (P3 or P5); however, the difference did not reach statistical significance (Fig. 8).

## Discussion

Mesenchymal stem cells (MSCs) have emerged as one of the most promising cell types for therapeutic applications due to their superior ability to repair injuries by generating cells of multiple differentiation lineages, their immunomodulation properties, and their potential for cancer therapy [28–30]. However, MSC properties vary significantly based on their tissue or organ of origin [11,31,32]. MSCs derived from human placenta (PMSCs) have recently gained increasing interest. The placenta is one of the most reliable and abundant sources for obtaining MSCs, and procurement of donor tissue is non-invasive and without major ethical concerns. Additionally, PMSCs have low immunogenicity, are relatively easy to expand *in vitro*, and have high differentiation plasticity [8,10,11,33]. Compared to MSCs derived from adult tissues, MSCs from fetal origin possess more therapeutic potential for both pre-clinical and clinical applications [5,9,10,34]. Indeed, our previous studies have demonstrated that human placental MSCs of fetal origin (fPMSCs) possess more therapeutic potential than cells of maternal origin or MSCs derived from other tissues [11].

Challenges remain persistence, however, as mounting evidence indicates that *in vitro* propagation of MSCs may cause deposition of genetic and/or epigenetic alterations [10,24,35]. In addition, the possibility that epigenetic alterations induce malignant transformation in MSCs is a major concern for applications in clinical settings. Currently, there is no solid evidence to support that MSCs undergo a spontaneous malignant transformation after extensive expansion *in vitro*, although several studies suggest that epigenetic mutations acquired during the course of propagation could induce malignancy [18,23]. Determining detrimental epigenetic alterations and how they may lead to tumorigenesis is therefore critical before the cells should be considered for therapeutic applications.

A great number of studies have revealed that MSCs might undergo spontaneous differentiation, aging, and/or replicative senescence after long-term culturing [10,24,36]. We have found previously that human fPMSCs acquire DNA methylation modifications during *in vitro* expansion in serum-free medium, but these epigenetic alterations do not confer malignancy in SCID mice [10].

In the present study, we analyzed modifications to histone acetylation in fPMSCs cultured in a serum-free medium. Although fPMSCs are able to sustain the morphology and immunophenotype of healthy MSCs after many passaged until they reach a senescent state, fPMSCs acquire alterations in epigenetic regulation with elevated HDAC enzymatic activity, increased expression of HDAC4, HDAC5 and HDAC6, and down-regulation of global histone H3/H4 acetylation during *in vitro* expansion. However, the *in vitro* propagation process did not significantly alter telomere length in fPMSCs. Intriguingly, consistent with findings from bone marrow-derived MSCs (BM-MSCs) cultured with a serum-free medium [37], the frequency of CD105-positive MSCs diminished gradually during the culturing process, suggesting that the serum-free medium might not provide an optimized microenvironmental niche for the maintenance of MSC stemness *in vitro*, even though the potential for generating multiple differentiation lineages was retained (data not show) [10].

Of note, fPMSCs entered a senescent state relatively quickly as shown by an increased fraction of β-galactosidase positive cells by P8 onwards in serum-free conditions [38], although they sustain a normal karyotype and telomere length during the cell expansion process [10]. However, mechanisms that effect biological behavior of cultured fPMSCs remain an important line of investigation.

Modifications to histones play key roles in regulation of gene expression and thusly regulate cell cycle progression and organogenesis. Histone acetylation/deacetylation alters chromosome structure, modulates the ability of transcription factors to access DNA. Histone acetylation/deacetylation can be catalyzed by histone acetyltransferase (HAT) or histone deacetylase (HDACs) activity. HDACs are a class of enzymes for histone acetylation modification, which is comprised of 18 members divided into 4 classes (class I, IIA/IIB, III and IV) based on their sensitivities to trichostatin A (TSA). Class I, II and IV HDACs consist of 11 members and are termed as canonical HDACs, and the other 7 members in class III are referred to as sirtuins [39,40].

Mounting evidence indicates that canonical HDACs play pivotal roles in cell metabolism, senescence, aging, carcinogenesis and other important biological processes [41,42], and several HDAC inhibitors have been developed for cancer therapy [43,44]. To date, although several lines of evidence suggest that the activity of HDACs is associated with properties of MSCs, such as cell proliferation, viability, differentiation, and aging [45–47], alterations of the HDAC profile and subsequent consequences to fPMSCs during *in vitro* expansion remain unclear. In this study, we found that global HDAC enzyme activity rather than a specific HDAC was significantly increased, along with an up-regulation of HDAC4, HDAC5 and HDAC6 during the culturing process using a serum-free medium. As a consequence, the level of global histone H3/H4 acetylation was strikingly decreased. These data imply that the transcriptional level of certain genes may be suppressed during long-term *in vitro* culture. Together with the notion that a high level of HDAC4, HDAC5 and HDAC6 expression has an implication in cancer development [48–50], the up-regulation of HDAC4, HDAC5 and HDAC6 of fPMSCs showed in this study may imply a potential risk for malignant transformation for this type of cells.

Li et al. recently found that genes associated with self-renewal, differentiation, and aging were modulated by histone acetylation in MSCs during *in vitro* propagation processes [24], however, the relationship between histone acetylations caused by alterations in HDAC activity and spontaneous malignant transformation of MSCs has not been established [19,20]. In addition, cytokines or chemical reagents in culture medium also had an impact on transformation of human MSCs into malignancy *in vitro* [23,51,52]. In this context, we therefore interrogated the expression of oncogenes Oct4, Sox2 and TERT in cultured fPMSCs over several passages, and the expression of these oncogenes was gradually down-regulated over time. Intriguingly, ChIP analysis only showed that reduced transcription of Oct4, but not Sox2 or TERT, could be related to significant changes to histone acetylation in its promoter region. This result suggests that other regulatory mechanisms may regulate the expression of Sox2 and TERT in fPMSCs. Given that Sox2 and TERT are critical regulators of self-renewal and multipotency in culture, further investigation should focus on mechanisms that contribute to their down-regulation in advanced-passage fPMSCs. The down-regulation of oncogenes, Oct4, Sox2, and TERT, during the *in vitro* propagation process may bestow biosafety to cultured fPMSCs used for clinical applications. However, our results slightly differed from a study by Li et al., which demonstrated that MSCs derived from placenta cultured in a medium supplemented with fetal bovine serum (FBS) could spontaneously diminish the expression of Oct4, Sox2, and TERT during long-term cultures, and this down-regulation was controlled by histone H3 acetylation [24]. Differences in media components between their study and ours may contribute to the apparent discrepancy, and the underlying mechanism by which specific media components may affect histone modifications in cultured MSCs remains to be investigated.

Sustaining long telomere length has been found to be a prominent property in multiple tissue-derived tumors [53,54], which provides tumor cells a selection bias for escaping cell senescence and thusly entering an immortal state of long-term proliferative potential [55–57]. This mechanism for maintaining telomere length is also adaptive for cultured MSCs, and the gradual shortening of telomeres has been observed in a diverse range of tissue-derived MSCs in long-term cultures before senescence and/or apoptosis programs are launched [58].

Previous studies have demonstrated that MSCs transfected with TERT have a greater potential for neoplastic, implying that telomerase activation or the status of telomeres may play an essential role for malignant transformation of MSCs [59,60]. Of note, in contrast MSCs generated from rat tissues, human MSCs normally contain much shorter telomeres. Indeed, freshly isolated human MSCs have shorter telomeres than in mice, thus short telomeres are not simply a result of diminished telomere maintenance in cultured cells [61]. In addition, human MSCs maintain a weaker telomerase activity than rat MSCs. These characteristics may contribute to why human MSCs are less prone to spontaneous malignant transformation over the course of *in vitro* propagation [62]. In agreement with this notion, shorter telomere lengths were detected in fPMSCs cultured in serum-free medium compared to MSCs from other tissue origins and/or culture conditions [17,23]. Moreover, telomere length gradually shortened over an extended culturing process, although this shortening was not statistically significant between passage numbers. Equally noteworthy, such shortening telomere may be one reason of the accelerated senescence of fPMSCs since the MSCs undergo a deposition of epigenetic alterations that modulate the expressions of many aging-related genes over the propagation process. These results may suggest that fPMSCs are less likely to develop spontaneous malignant transformation than to enter replicative senescence when they are cultured in serum-free conditions. This notion is supported by our previous study investigating tumorigenicity potential of fPMSCs in SCID mice (data no show) [10].

Collectively, in this report, we investigated alterations in acetylation and subsequent epigenetic consequences of human fPMSCs during *in vitro* expansion in serum-free culture conditions. Our results demonstrate that cultured fPMSCs acquire enhanced global HDAC activity, up-regulate the expression of HDAC4, HDAC5 and HDAC6, and display a down-regulated global histone H3/H4 acetylation pattern as a result of an *in vitro* expansion process using serum-free medium. The expression of oncogenes Oct4, Sox2, and TERT were significantly decreased over long-term culture, but only the down-regulation of Oct4 could be strongly associated with a change in acetylation. In addition, we demonstrate that telomere length of fPMSCs does not significantly shorten during *in vitro* propagation. These findings imply that fPMSCs are a stable and safe source for therapeutic MSCs and possess less potential to develop spontaneous malignancy when they are expanded in serum-free conditions and warrants further validation in pre-clinical and clinical settings.
